# Proteomics reveals proteins linked to the quality of wood in contrasting xylem of *Eucalyptus* clones

**DOI:** 10.1186/1753-6561-5-S7-P155

**Published:** 2011-09-13

**Authors:** Dahyana Britto, Carlos Pirovani, Esteban Gonzalez, João Silva, Abelmon Gesteira, Júlio Cascardo

**Affiliations:** 1 Center of Biothecnology and Genetics, Dep. of Genetics and Molecular Biology, Universidade Estadual de Santa Cruz - UESC, Ilhéus, BA, 45662-900, Brazil; 2 Suzano Paper and Cellulose S.A., Mucuri, BA, 45936-000, Brazil; 3 Brazilian Enterprise for Agricultural Research cassava and fruit - EMBRAPA, , Cruz das Almas, BA, 44380-000, Brazil; 4 in memorian

## Background

Eucalyptus spp. is a genus widely planted in many tropical and subtropical regions of the world as a major source in the production of pulp and paper. Aiming to meet the demand required by the world market papermaker, with the need for greater productivity in forests, as well as higher quality of wood, reducing the amount of lignin and extractive, is that proteomics emerges as an additional tool to help accelerate progress in the selection of elite clones.

## Materials and methods

In this study, using the combination of the two-dimensional gel electrophoresis (2D-PAGE) and electrospray mass spectrometry on quadrupole-time of flight (ESI-QUAD-TOF) identify differentially expressed proteins between four genotypes of eucalyptus, brothers siblings, contrasting with differences for components of wood quality. The components evaluated in this study for four xylems were: wood density, which was higher in clones X1 and X2, total lignin content, with levels higher in the X3 and X4 clones, extractive content, with higher values for the clones X2 and X4, and gross yield in pulp, which was higher in clones X1 and X3. Whereas the desirable characteristics for application in pulp and paper industry is the clone that best combines the highest density of wood and pulp yield, with lower lignin content and extractive, the X1 is the clone that best meets these criteria, the most suitable for such application, and X4 clones, considered inadequate for the same process.

## Results

The profile followed by 2D gel analysis software with 3D Image Master Platinum xylem protein extracts of these clones resulted in identification of 30 spots differentially expressed, with 3 proteins in clone X1, 4 proteins in clone X2, 11 proteins in clone X3 and 12 proteins in clone X4. These differential spots were excised, subjected to tryptic digestion and processing, followed by mass spectrometry analysis.The identified proteins are involved in various biological processes, including polyphosphate biosynthetic process, catalytic activity, nucleotide-excision repair, cellular metabolic process, cell redox homeostasis, response to salt stress, response to heat, oxidation-reduction process, potassium ion transport, electron transport chain, response to wounding, superoxide metabolic process, response to water deprivation, protein phosphorylation. It is noteworthy that the proteins involved in oxidative stress were up regulated in clone X4, followed by clones X2 and X3, and to a lesser extent in clone X1. Among the proteins differentially expressed in clone X4 can highlight catalase, superoxide dismutase, annexins, heat shock protein (chaperone) and ABC transporter. Catalase is an enzyme that converts 2H2O2 in O2 + 2H2O, primarily by preventing the potential damage caused by changes in H2O2 homeostasis [1]. The superoxide dismutase is the first line of plant defense against reactive species of O2, O2- removing the cellular compartments where this radical is formed [2]. Annexin peroxidase may play a role as the process of oxidative stress in plants [3]. The first reviews of plant ABC transporter showed that they participate in detoxification processes, but evidence already exists of their participation in the flow of ions, and being involved in the process of growth and development of plants [4]. The molecular chaperones known as heat shock proteins (Hsp) are stress proteins. Under stress conditions, Hsp facilitates protein folding and help stabilize polypeptides and membranes [5].

## Conclusions

The proteins identified in the study involved in redox process in the plant, as well as the extractives, including polyphenols, tannins and resins, components characterized as inadequate in the pulp and paper industry, are induced as a defense response to pathogen attacks as well as to abiotic stress [1,6]. Furthermore, it has been demonstrated that oxidative stress can also induce lignification in plant tissue [7,8].Given this, we infer that the presence of proteins involved in oxidative stress in the xylem of a eucalyptus is a strong predictor of inappropriate clones in the pulp and paper industry.

Selection of candidate genes and validation study of gene expression through genetic materials in the wood quality will be known the next step in the selection of genetic markers to identify elite clones of eucalyptus for the pulp and paper production.
